# Interaction between the inflammasome and commensal microorganisms in gastrointestinal health and disease

**DOI:** 10.15252/emmm.202013452

**Published:** 2021-10-27

**Authors:** Daisuke Watanabe, Yijie Guo, Nobuhiko Kamada

**Affiliations:** ^1^ Division of Gastroenterology and Hepatology Department of Internal Medicine University of Michigan Ann Arbor MI USA

**Keywords:** gut microbiota, inflammasome, interleukin 18, interleukin 1β, Immunology, Microbiology, Virology & Host Pathogen Interaction

## Abstract

The inflammasome is a cytosolic multiprotein complex that plays a crucial role in inflammation and cell death. The sensor proteins in the inflammasome complex detect various microbial and endogenous stimuli, leading to subsequent caspase activation. The activation of caspases results in the maturation of pro‐inflammatory cytokines IL‐1β and IL‐18 or pyroptosis. Inflammasome dysfunction is associated with the pathogenesis of various diseases, including autoimmune disease and cancer. It appears that the interactions between the gut microbiota and the inflammasome play crucial roles in the gastrointestinal tract. The gut microbiota induces the expression and activation of inflammasome proteins, which contribute to both homeostasis and disease in the gut. Likewise, although controversial, mounting evidence suggests that inflammasome activation can modulate the composition of the gut microbiota, which, in turn, affects disease progression. In this review, we summarize the current concepts and recent insights linking the inflammasome and gut commensal microorganisms. We describe how the reciprocal interaction between the inflammasome and the commensal microbiota relates to physiological and pathophysiological consequences in the host.

## Graphical abstract

In this review, N. Kamada and colleagues discuss the effects of the crosstalk between the inflammasome and commensal microorganisms on the physiology and pathophysiology of the host.

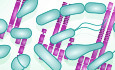

**Glossary**
Antimicrobial peptides: Antimicrobial peptides (AMPs) are synthesized natural antibiotics produced by nearly all organisms, from bacteria and archaea to plants and animals.
Commensal bacteria: “Commensal” originates from the Latin meaning “sharing the same table”, an alternative term for microbiota. Commensal bacteria are defined as the normal beneficial gut resident bacteria of importance for digestion of food and protecting the gut against pathogenic bacteria.
Damage‐associated molecular patterns (DAMPs): Intracellular molecules released from damaged or dying cells that can activate the innate immune system through their interaction with pattern recognition receptors (PRRs.)
Dysbiosis: Dysbiosis is the condition that describes an imbalance of beneficial and pathogenic bacteria in the microbiota, and it has a negative impact on the physiology of the host.
Inflammasome: Multimeric protein complex consists of an inflammasome sensor, the adaptor protein ASC, and cysteine protease caspase‐1. Its primary function is to process pro‐IL‐1β and pro‐IL‐18 into their mature forms and to execute inflammatory cell death termed pyroptosis.
Inflammatory bowel disease (IBD): A group of intestinal disorders characterized by chronic inflammation of the bowel. Ulcerative colitis (UC) and Crohn's disease (CD) are the two most common types of IBD.
Lipoteichoic acid (LTA): LTA is a cell wall component exclusive to Gram‐positive bacteria and is shed during bacterial replication and after therapeutic administration of antibiotics. LTA is considered an immunostimulatory functional equivalent to lipopolysaccharide (LPS), a major cell wall component of Gram‐negative bacteria.
Pathogen‐associated molecular patterns (PAMPs): Conserved molecular structures shared by several pathogens that are recognized by innate immune receptors.
Pyroptosis: A gasdermin D (GSDMD)‐dependent programmed cell death is initiated by caspase‐1 or caspase‐11 activation. It occurs most frequently on infection with intracellular pathogens.
Toll‐like receptors (TLRs): Germline‐encoded receptors recognize pathogen‐associated molecular patterns. TLR signals result in the activation of cells and the production of pro‐inflammatory mediators.

## Introduction

The term inflammasome was introduced in 2002 to describe an inducible high molecular weight complex containing nucleotide‐binding domain, leucine‐rich repeat containing protein (NLR) family pyrin domain containing 1 (NLRP1), PYCARD (ASC), and caspase‐1. Consistent with the NLRP1 inflammasome, an inflammasome complex usually consists of three components: a sensor component, an adapter molecule, and an effector component. To date, several kinds of inflammasomes have been identified and broadly classified into three groups based on the sensor component: NLR‐associated inflammasomes, absent in melanoma‐2 (AIM2)‐like receptor (ALR)‐associated inflammasomes, and the pyrin inflammasome (Vanaja *et al*, 2015; Broz & Dixit, 2016). Inflammasomes can be activated by a variety of pathogen‐associated molecular patterns (PAMPs) and damage‐associated molecular patterns (DAMPs). The inflammasome activation by PAMPs and DAMPs leads to the activation of caspase‐1, thereby cleaving pro‐forms of interleukin (IL)‐1β and IL‐18 to generate mature forms of these cytokines (Schroder & Tschopp, 2010; Vanaja *et al*, 2015; Broz & Dixit, 2016). Moreover, inflammasome activation can cause pyroptosis, which is a form of inflammatory cell death.

The gastrointestinal tract is continuously exposed to a dense microbial community, called the microbiota (Sender *et al*, 2016). The commensal microbiota modifies numerous aspects of host physiology and pathophysiology, including complex mutual interactions with the host's immune system (Medzhitov, 2007; Gensollen *et al*, 2016). Inflammasome proteins are expressed in both immune (e.g., macrophages, dendritic cells) and non‐immune cells (intestinal epithelial cells, fibroblasts) (Kummer *et al*, 2007; Elinav *et al*, 2011). Given their role as microbial sensors (Zheng *et al*, 2020), the activation of inflammasomes by the commensal microbiota may contribute to physiological and pathophysiological consequences in the host (Strowig *et al*, 2012).

Herein, we review current concepts and recent insights linking the inflammasome and commensal microorganisms. We highlight the mechanisms by which the inflammasome and commensal microbiota regulate each other, and how the reciprocal interaction between the inflammasome and commensal microbiota relates to physiological and pathophysiological consequences in the host.

## NLRP1 inflammasome

NLRP1 was the first NLR shown to form an inflammasome complex by recruiting ASC and caspase‐1 (Martinon *et al*, 2002). Polymorphisms of human NLRP1 are associated with increased risk for many diseases, such as vitiligo (Jin, Birlea, *et al*, 2007; Jin, Mailloux, *et al*, 2007), Addison's disease (Magitta *et al*, 2009; Zurawek *et al*, 2010), type 1 diabetes (Magitta *et al*, 2009), and systemic lupus erythematosus (Pontillo *et al*, 2012). In addition, the NLRP1 inflammasome has been considered to be an important mediator, maintaining the host intestinal microbiota and controlling intestinal pathophysiology, as occurs in response to inflammatory bowel disease (IBD) (Cummings *et al*, 2010; De Iudicibus *et al*, 2011). Genome‐wide association studies (GWASs) have identified NLRP1 mutations associated with Crohn’s disease or its extraintestinal manifestations, such as erythema nodosum and pyoderma gangrenosum (Cummings *et al*, 2010). Further, a retrospective analysis of metadata from colonic mucosal biopsies collected from patients with active ulcerative colitis (UC) revealed a higher expression level of NLRP1 (Williams *et al*, 2015).

**Figure 1:**
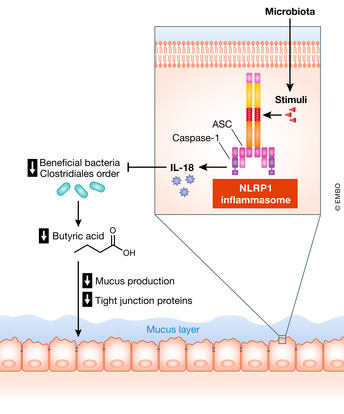
NLRP1 inflammasome may contribute to intestinal inflammation through IL‐18 The NLRP1 inflammasome suppresses beneficial bacteria through IL‐18. IL‐18, likely via the induction of antimicrobial peptides (AMPs), inhibits the growth of butyrate‐producing bacteria, such as species in the order Clostridiales. The reduced population of butyrate‐producing bacteria impairs intestinal barrier functions (e.g., mucus production, expression of tight junction proteins).

In an animal model of IBD, it has been reported that NLRP1 exacerbates colitis through the interaction with commensal microbes (Fig 1). Tye and colleagues revealed that the NLRP1 inflammasome modulates the gut microbiota of littermate control mice (Tye *et al*, 2018). Mice deficient in *Nlrp1* display an increased abundance of butyrate‐producing bacteria of the order Clostridiales, which protects against DSS‐induced colitis. Butyrate has been shown to have beneficial effects on IBD pathologies by enhancing intestinal barrier functions, including mucus production and the expression of tight junction proteins (Van Immerseel *et al*, 2010). Therefore, the NLRP1 inflammasome may have a negative impact on IBD through reducing butyrate production by the gut microbiota. How does the NLRP1 inflammasome modulate the composition of the gut microbiota? In this context, IL‐1β signaling is dispensable for the microbiota modulation by NLRP1. Instead, IL‐18 activation by NLRP1 contributes to DSS‐induced colitis phenotype (Tye *et al*, 2018). Consistent with the negative impact of IL‐18 on butyrate‐producing bacteria in mice, the expression level of IL‐18 in human intestinal biopsy samples reveals a negative correlation with the population of Clostridiales (Tye *et al*, 2018). NLRP1 is expressed in various organs, including glandular epithelial structures such as the stomach, intestine, lung, nerve, and testis (Kummer *et al*, 2007). At the cellular level, NLRP1 is expressed in a broad array of cell types, including granulocytes, monocytes, dendritic cells, and B and T cells (Kummer *et al*, 2007). Among these cells, it has been shown that non‐hematopoietic compartments are responsible for the exacerbation of DSS‐induced colitis (Tye *et al*, 2018).

In contrast to the colitogenic role of NLRP1, there is evidence that the NLRP1 inflammasome attenuates intestinal inflammation (Williams *et al*, 2015). Williams and colleagues reported that *Nlrp1b*‐deficient mice are more susceptible to DSS‐induced colitis than wild‐type (WT) littermates (Williams *et al*, 2015). Given that the treatment with antibiotics reversed this phenotype in *Nlrp1b*‐deficient mice, it is plausible that the increased susceptibility to colitis is through the modulation of microbiota caused by the loss of NLRP1b (Williams *et al*, 2015). Likewise, co‐housing with *Nlrp1b*‐deficient mice can transmit the susceptible phenotype to WT mice (Williams *et al*, 2015). Thus, NLRP1 deficiency induces the expansion of colitogenic bacteria. Note, however, that the impact of the genotype of the NLRP1 allele is weaker than maternal influence when comparing littermates bred from homozygous and heterozygous parents (Ringel‐Scaia *et al*, 2019). As in the contradictory report by Tye and colleagues, a bone marrow chimeric experiment revealed that the NLRP1 inflammasome functions in a non‐hematopoietic compartment. Several possibilities may explain this discrepancy. One possibility is the genetic variability of the mouse strains. Unlike humans, mice possess three paralogs of *Nlrp1* (a, b, and c) (Sastalla *et al*, 2013). The mice used in the study were *Nlrp1* deficient, lacking all three alleles of *Nlrp1*, whereas the *Nlrp1b*‐deficient mice used by Williams and colleagues retain a functional *Nlrp1a*. Another possibility is a difference in the cytokines associated with disease phenotype. In contrast to the results reported by Tye and colleagues, the Williams laboratory observed that both IL‐1β and IL‐18 are responsible for the attenuation of colitis. Collectively, these two studies show that the NLRP1 inflammasome has the physiological capability to influence the composition of the intestinal microbiota through IL‐18 and/or IL‐1β. However, further study is necessary to reveal the functional consequence of NLRP1‐mediated modulation of the commensal microbiota.

## NLRP3 inflammasome

The NLRP3 inflammasome consists of three basic elements: NLRP3, ASC, and procaspase‐1 (Agostini *et al*, 2004). The NLRP3 inflammasome can be activated by a two‐step mechanism in response to numerous diverse stimuli derived from microbial and endogenous molecules (Franchi, Munoz‐Planillo, *et al*, 2012). In the first step, termed the priming, certain PAMPs or DAMPs induce the expression of NLRP3 and pro‐form of IL‐1β in targeted cells (Franchi, Munoz‐Planillo, *et al*, 2012). In the second step, the ligands for NLPR3, such as ATP or the bacterial toxin nigericin, elicit the oligomerization of NLRP3, followed by the assembly of NLRP3, ASC, and procaspase‐1 into the NLRP3 inflammasome complex (Franchi, Munoz‐Planillo, *et al*, 2012). In addition to these well‐known mechanisms of NLRP3 activation, posttranscriptional regulation through the ubiquitin–proteasome system has been reported in large intestinal‐resident macrophages (Filardy *et al*, 2016). Uncontrolled activation of NLRP3 is known to be associated with the pathogenesis of various inflammatory diseases in humans. For example, gain‐of‐function mutations in the NLRP3 gene are linked to cryopyrin‐associated periodic fever syndromes (CAPS), a group of rare hereditary autoinflammatory diseases, including familial cold urticaria, Muckle–Wells syndrome, and neonatal onset multisystem inflammatory disease (Hoffman *et al*, 2001; Hoffman & Wanderer, 2010). In addition, a case–control study suggested the association between NLRP3 and intestinal inflammation. The NLRP3 rs10754558 single‐nucleotide polymorphism (SNP) is as a gain‐of‐function mutation (Hitomi *et al*, 2009) that is associated with ulcerative colitis, as the GG genotype of rs10754558 was 2.48 times more common among patients with this disease (Hanaei *et al*, 2018).

**Figure 2:**
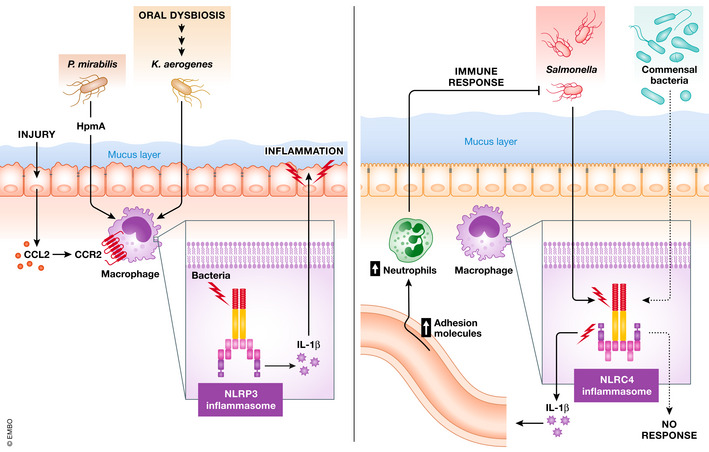
The dialogue between intestinal microbiota and physiological response via NLRP3 and NLRC4 inflammasomes The activation of the NLRP3 inflammasome and subsequent IL‐1β production result in the exacerbation of intestinal inflammation (left panel). Hemolysin produced by commensal *Proteus mirabilis* activates the NLRP3 inflammasome in CCR2^+^Ly6C^high^ newly recruited monocytes through hemolysin A (HpmA). Likewise, ectopically colonized oral pathobionts, such as *Klebsiella aerogenes*, activate the NLRP3 inflammasome in intestinal macrophages. In contrast, the NLRC4 inflammasome behaves as a gatekeeper for intestinal pathogens (right panel). Commensal bacteria fail to activate the NLRC4 inflammasome in intestinal macrophages, whereas enteric pathogens, such as *Salmonella*, succeed. Pathogen‐elicited inflammasome‐mediated IL‐1β secretion, in turn, promotes the eradication of the pathogens through activation of the host's innate immune defense (i.e., neutrophil recruitment via the induction of adhesion molecules in the endothelial cells).

Several reports show that the NLRP3 inflammasome modulates the intestinal microbiota (Zaki *et al*, 2010; Hirota *et al*, 2011; Yao *et al*, 2017). Recognizing the need for standardized littermate‐controlled experimental design to investigate host genetics and intestinal microbial interaction, Hirota and colleagues and Yao and colleagues used Nlrp3‐deficient mice and their WT littermate controls. In contrast, the role of commensal microbes in NLRP3 activation in the context of the pathogenesis of inflammatory diseases is studied extensively in animal models (Fig 2). In the CAPS mouse model Nlrp3^R258W^, skin commensal microbes induce the activation of the NLRP3 inflammasome, thereby promoting cutaneous inflammation (Nakamura *et al*, 2012). Certain commensal pathobionts in the gut are reported to be capable of activating the NLRP3 inflammasome in mononuclear phagocytes residing in the intestinal mucosa. Seo and colleagues identified the gut pathobiont *Proteus mirabilis* as a potential activator of NLRP3 (Seo *et al*, 2015). *Proteus mirabilis* produces hemolysin, which plays a central role in the induction of IL‐1β by macrophages through the activation of the NLRP3 inflammasome (Seo *et al*, 2015). The NLRP3 activation by *P. mirabilis* is critical for the exacerbation of colitis caused by the colonization of this bacterium (Seo *et al*, 2015). More recently, Kitamoto and colleagues demonstrated that *Klebsiella* and *Enterobacter* species residing in the oral cavity are capable of activating the NLRP3 inflammasome (Kitamoto *et al*, 2020). For example, *K. aerogenes*, which accumulates in the oral cavity of mice that develop periodontitis, induces a robust secretion of IL‐1β by macrophages through the activation of NLRP3 (Kitamoto *et al*, 2020). Importantly, ingested *K. aerogenes* reaches the gastrointestinal tract, and the ectopic gut colonization by this bacterium exacerbates intestinal inflammation in DSS‐induced colitis and *Il10*‐deficient mice (Kitamoto *et al*, 2020). Similar to *P. mirabilis*, the lack of NLRP3 or the blockade of IL‐1 signaling cancels the colitogenic effect of *K. aerogenes*, suggesting the central role of the NLRP3–IL‐1β axis in the pathogenesis of oral commensal pathobiont‐driven colitis (Kitamoto *et al*, 2020). Likewise, IL‐18, induced as a mediator downstream of the NLRP3 inflammasome activation, impairs intestinal barrier integrity through inhibition of goblet cell maturation (Nowarski *et al*, 2015). Thus, mounting evidence supports the notion that the NLRP3 inflammasome and its downstream mediators, such as IL‐1β and IL‐18, play a central role in the induction and exacerbation of intestinal inflammation (Bauer *et al*, 2010; Seo *et al*, 2015). However, several studies report the opposite results. For example, NLRP3 inflammasome activation in epithelial cells by GPCR signaling contributes to protection from DSS‐induced colitis (Macia *et al*, 2015). Zaki and colleagues showed that mice lacking *Nlrp3* are highly susceptible to DSS‐induced colitis (Zaki *et al*, 2010), which is consistent with some other reports (Allen *et al*, 2010; Hirota *et al*, 2011). These investigators observed that NLRP3 activation leads to the secretion of IL‐18, which protects the host from colitis (Zaki *et al*, 2010). Likewise, *Il18*‐ and *Il18r1*‐deficient mice display increased susceptibility to DSS‐induced colitis (Takagi *et al*, 2003). IL‐18 acts on intestinal epithelial cells and promotes barrier integrity (Zaki *et al*, 2010). Moreover, IL‐18 signaling modulates the commensal microbiota to be more homeostatic by suppressing the population growth of pathobionts (Zaki *et al*, 2010). This discrepancy may, in part, be due to differences in the gut microbiota. For example, some of these studies used non‐littermate controls, and it is possible that the knockout mice were WT control mice with a different microbial composition (Takagi *et al*, 2003; Allen *et al*, 2010; Zaki *et al*, 2010). In this context, it has been reported that inflammasome activation is detrimental if the microbiota is normalized before the induction of colitis (Blazejewski *et al*, 2017). Thus, despite enduring controversy, the activation of the NLRP3 inflammasome by certain pathobionts may play a crucial role in the development or exacerbation of intestinal inflammation.

## NLRC4 inflammasome

NLRC4 was first described in 2001 as a mammalian protein homologous to CED4 of *Caenorhabditis elegans* that recruits and activates caspases through its CARD domain (Geddes *et al*, 2001; Poyet *et al*, 2001). Different from other NLR family members, the assembly and activation of the NLRC4 inflammasome requires interaction with NLR apoptosis inhibitory proteins (NAIPs) (Kofoed & Vance, 2011; Zhao *et al*, 2011). In mice, seven NAIP paralogs have been identified. Mouse NAIP1 and NAIP2 bind the needle and rod proteins of the type III secretion system (T3SS), whereas mouse NAIP5 and NAIP6 bind cytosolic flagellin (Kofoed & Vance, 2011; Zhao *et al*, 2011). In contrast, the only known human NAIP can detect T3SS needle protein and flagellin, but not T3SS rod protein (Zhao *et al*, 2011; Kortmann *et al*, 2015). The binding of a NAIP by its corresponding bacterial ligand induces a physical interaction between the NAIP and NLRC4, which leads to the formation of an oligomeric NLRC4 inflammasome complex. This complex serves as a platform for inducing caspase‐1 activation through a CARD–CARD interaction between NLRC4 and caspase‐1. As for the NLRC4 ligands, the activation of the NLRC4 inflammasome is known to be induced by Gram‐negative pathogens through the translocation of small amounts of flagellin or PrgJ‐like rod proteins (T3SS rod proteins) into the host cytosol (Franchi *et al*, 2006; Miao *et al*, 2010). Thus, in macrophages, NLRC4 can be activated by cytosolic flagellin derived from *Salmonella enterica* serovar Typhimurium (*Salmonella*) (Franchi *et al*, 2006). In addition to *Salmonella*, NLRC4 is capable of identifying other Gram‐negative bacteria that possess either flagellin or T3SS factors, including *Pseudomonas aeruginosa* (Sutterwala *et al*, 2007), *Legionella pneumophila* (Zamboni *et al*, 2006), and *Shigella flexneri* (Suzuki *et al*, 2007). As for the pathophysiological role of NLRC4, clinical studies have shown that gain‐of‐function NLRC4 mutations are associated with autoinflammation with infantile enterocolitis (AIFEC) (Canna *et al*, 2014; Romberg *et al*, 2014), an extremely rare disease that is characterized by macrophage activation syndrome and severe inflammation of gastrointestinal tract.

Notably, the NLRC4 inflammasome also has the ability to discriminate certain intestinal bacteria from others, akin to the NLRP3 inflammasome (Fig 2) (Franchi, Kamada, *et al*, 2012; Seo *et al*, 2015). The bacteria discriminating function of the NLRP3 inflammasome is evident in inflammatory monocytes, whereas the NLRC4 inflammasome is expressed in resident intestinal mononuclear phagocytes (iMPs), including macrophages and dendritic cells, and reacts to pathogenic microbes while maintaining tolerance to commensal microbes (Franchi, Kamada, *et al*, 2012). As immune cells in the intestine are continuously exposed to a large number of commensal microbes, iMPs harbor mechanisms that limit excessive immune responses against commensal microbes. Indeed, iMPs are not activated by bacterial molecules, such as TLR ligands, derived from commensals, nor do they secrete pro‐inflammatory cytokines, such as tumor necrosis factor (TNF)‐α or IL‐6 (Smythies *et al*, 2005; Lotz *et al*, 2006; Franchi, Kamada, *et al*, 2012). In contrast, iMPs constitutively express pro–IL‐1β and NLRC4 (Franchi, Kamada, *et al*, 2012). However, unlike other pro‐inflammatory cytokines, such as TNF‐α and IL‐6, the pro‐form of IL‐1β is not biologically active, nor does it induce inflammation. As the commensal bacteria fail to activate the NLRC4 inflammasome, iMPs do not secrete mature, bioactive IL‐1β when stimulated by these bacteria (Franchi, Kamada, *et al*, 2012). On the other hand, iMPs need to respond if stimulated by pathogenic microbes to provoke antimicrobial immunity to combat the pathogens. In this regard, iMPs can secrete IL‐1β in response to pathogenic microbes, such as *Salmonella*, which activate NLRC4 inflammasome (Franchi, Kamada, *et al*, 2012). Thus, iMPs discriminate pathogens from commensal bacteria through the NLRC4 inflammasome sensor. IL‐1β secreted from iMPs as a result of NLRC4 activation stimulates endothelial cells to express adhesion molecules, such as VCAM‐1, ICAM‐2, E‐selectin, and P‐selectin, which enhance the recruitment of neutrophils to the intestinal mucosa and eradicate invasive pathogens (Franchi, Kamada, *et al*, 2012). Similarly, the NLRC4 inflammasome intrinsic to intestinal epithelial cells restricts *Salmonella* replication using a mechanism that drives infected enterocyte expulsion (Sellin *et al*, 2014). Moreover, NLRC4 inflammasome activation by commensal bacteria confers disease tolerance on the host. Schieber and colleagues revealed that colonization by the specific commensal *Escherichia coli* O21:H^+^ strain, which is resistant to a broad spectrum of antibiotics including ampicillin, vancomycin, neomycin, and metronidazole (namely AVNM^R^ *E. coli*), mitigates DSS‐induced colitis, *Salmonella*‐induced colitis, and *Burkholderia thailandensis*‐induced pneumonia (Schieber *et al*, 2015). Colonization by the *E. coli* O21:H^+^ strain induces insulin/insulin‐like growth factor‐1 (IGF‐1) through the activation of NLRC4, which plays a central role in disease tolerance in these models (Schieber *et al*, 2015). How the microbiota induces IGF‐1 systemically and locally remains under investigation. Although IGF‐1 is mainly regulated by growth hormone, previous reports have shown that short‐chain fatty acids (Yan *et al*, 2016) and amino acids (Takenaka *et al*, 2000) can modulate IGF‐1 production. This suggests that NLRC4 activation accelerates host IGF‐1 production by way of metabolites produced by the microbiota.

## NLRP6 inflammasome

The initial study of the NLR family pyrin domain containing protein 6 (NLRP6/PYPAF5) in 2002 demonstrated that co‐expression of NLRP6 and ASC in human cell lines results in the activation of caspase‐1 and NF‐κB (Grenier *et al*, 2002). Despite contradictory evidence that NLRP6 negatively regulates NF‐κB signaling (Anand *et al*, 2012), NLRP6 is unique in the NLR family as it can behave like both NLRs and Toll‐like receptors (TLRs). Structurally, NLRP6 comprises an N‐terminal PYD, a central NACHT domain, and a C‐terminal leucine‐rich repeat (LRR) domain, similar to the NLRP3 protein. Further, NLRP6 and NLRP3 proteins share similarities in amino acid sequences—32% in humans and 33% in mice. However, NLRP6 and NLRP3 recognize different ligands given the variations in their C‐terminal LRR domains (Li & Zhu, 2020). It has been reported that bacterial infection induces NLRP6 co‐localization with ASC in the cytoplasm of bone marrow‐derived macrophages, thereby leading to the activation of caspase‐1 and the secretion of mature IL‐1β (Grenier *et al*, 2002; Ghimire *et al*, 2018). Hara and colleagues showed that lipoteichoic acid (LTA) from Gram‐positive bacterial pathogens, such as *Listeria monocytogenes*, induces the expression of NLRP6 and caspase‐11 via type I interferon (IFN) signaling. LTA also serves as a ligand, binding to NLRP6 to activate the inflammasome via the ASC–caspase‐11–caspase‐1 signaling cascade (Hara *et al*, 2018).

**Figure 3:**
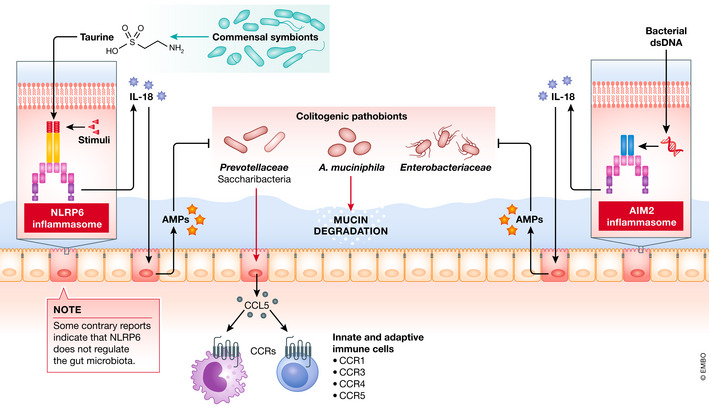
The dialogue between intestinal microbiota and physiological response via NLRP6 and AIM2 inflammasomes The NLRP6 inflammasome modulates the gut microbiota and maintains gastrointestinal homeostasis. NLRP6‐dependent production of IL‐18 contributes to the secretion of antimicrobial peptides (AMPs) from intestinal epithelial cells. IL‐18, likely through AMPs, suppresses the growth of colitogenic pathobionts, such as bacteria in the family *Prevotellaceae* and the phylum Saccharibacteria (formerly known as TM7), whose colonization induces CCL5‐mediated recruitment of inflammatory cells. Likewise, the NLRP6–IL‐18 axis is crucial for the regulation of the colonization of *Akkermansia muciniphila*. Hence, the NLRP6 inflammasome maintains intestinal barrier function by suppressing mucin degradation by *A. muciniphila*. Of note, some contrary reports indicate that NLRP6 deficiency has no influence on the composition of the gut microbiota. Thus, the interaction between the NLRP6 and the gut microbiota remains controversial. On the other hand, the gut microbial metabolite taurine induces NLRP6 expression. Like NLRP6, the AIM2 inflammasome suppresses the growth of potential pathobionts, such as *Enterobacteriaceae*, through the IL‐18–AMP axis.

To date, most studies of NLRP6 have concentrated on defining its role in the gastrointestinal tract, where it is primarily expressed (Elinav *et al*, 2013). It has been repeatedly demonstrated that the NLRP6 inflammasome is associated with the homeostatic maintenance of the gastrointestinal tract (Fig 3). Accumulating evidence indicates that (i) dysregulation of the NLRP6 inflammasome results in intestinal dysbiosis (Elinav *et al*, 2011; Seregin *et al*, 2017), (ii) the NLRP6 inflammasome can induce the production of antimicrobial peptides (AMPs) (Levy *et al*, 2015), and (iii) loss of the NLRP6 inflammasome results in autophagy dysfunction (Wlodarska *et al*, 2014). Elinav and colleagues showed that the fecal microbiota of non‐littermate *Nlrp6*‐deficient mice consists of distinct bacterial communities with a predominance of species from the phylum Saccharibacteria (formerly known as TM7) and the family *Prevotellaceae* (Elinav *et al*, 2011). Of note, the transfer of the dysbiotic microbiota from *Nlrp6*‐deficient mice to WT mice confers susceptibility to DSS‐induced colitis, suggesting that NLRP6 may be crucial for the fine‐tuning of the gut microbiota. An aberrant gut microbiota caused by NLRP6 deficiency results in the expression of CCL5 in the intestinal epithelial cells (Elinav *et al*, 2011). CCL5 induces the recruitment of a variety of innate and adaptive immune cells carrying CCR1, CCR3, CCR4, and CCR5 (Mantovani *et al*, 2004). The elevation of CCL5, in particular, renders the host susceptible to colitis. Similarly, Seregin and colleagues showed that deficiency in NLRP6 increases the susceptibility to colitis in *Il10* ^−/−^ mice (Seregin *et al*, 2017). Consistent with the report from Elinav’s laboratory, NLRP6 deficiency leads to gut dysbiosis with the expansion of *Akkermansia muciniphila*, a Gram‐negative and strictly anaerobic bacterium that belongs to the phylum Verrucomicrobia. *Akkermansia muciniphila*, a member of the resident gut microbiota in mice and humans, is capable of degrading mucin (Derrien *et al*, 2004). Although it remains controversial whether *A. muciniphila* serves as the pathobiont in intestinal inflammatory diseases, such as IBD (Png *et al*, 2010; Kang *et al*, 2013; Rajilic‐Stojanovic *et al*, 2013), excessive mucin degradation by *A. muciniphila* clearly impairs the intestinal mucus barrier. Indeed, the gut colonization by *A. muciniphila* aggravates colitis in *Il10* ^−/−^ mice (Seregin *et al*, 2017). Similarly, Sun and colleagues revealed that inhibition of Nlrp6 expression by corticotropin‐releasing hormone leads to intestinal inflammation and dysbiosis (Sun *et al*, 2013).

Multiple mechanisms have been proposed to explain how NLRP6 modulates the gut microbiota. Seregin and colleagues demonstrated that NLRP6‐dependent production of IL‐18 is responsible for the regulation of the gut microbiota. Both *Il18* ^−/−^ and *Il18r1* ^−/−^ mice display an over representation of *A. muciniphila*, and the administration of recombinant IL‐18 reduces the abundance of *A. muciniphila* in *Nlrp6* ^−/−^ mice (Seregin *et al*, 2017). IL‐18 plays a key role in host defense by regulating the intestinal mucus barrier (Nowarski *et al*, 2015). Of these mechanisms, intestinal mucin produced by goblet cells may affect the abundance of *A. muciniphila* (Desai *et al*, 2016). Although *Nlrp6* ^−/−^ mice display impaired maturation of goblet cells (Wlodarska *et al*, 2014), IL‐18 inhibits goblet cell maturation, which can lead to a thinner mucus layer (Nowarski *et al*, 2015). This suggests that alteration of *A. muciniphila* abundance may be attributed to the promoted mucus production. Note, however, that NLRP6 inflammasome function alone does not influence mucus production, as reported in studies that use proper littermate control animals (Volk *et al*, 2019). In addition to its influence on the mucus layer, IL‐18 may modulate the abundance of *A. muciniphila* through the induction of antimicrobial peptides (AMPs) (Levy *et al*, 2015). It has been reported that IL‐18 signaling regulates the expression of AMPs, including angiogenin‐4 (Ang4), in the gastrointestinal tract (Levy *et al*, 2015). AMPs can modulate the intestinal microbiota composition (Vaishnava *et al*, 2011; Cullen *et al*, 2015). Given that appropriate AMP secretion by intestinal epithelial cells is crucial for the maintenance of intestinal homeostasis (Ostaff *et al*, 2013; Mukherjee & Hooper, 2015), IL‐18 may play a central role in reshaping the composition of the gut microbiota by the NLRP6 inflammasome. In this regard, the injection of Ang4 into mice deficient in ASC, a component of the NLRP6 inflammasome complex, restores the gut microbial community (Levy *et al*, 2015). Notably, the gut resident microbiota can also act upstream of NLRP6. Microbial metabolites, such as taurine, histamine, and spermine, appear to activate NLRP6‐dependent secretion of IL‐18 and subsequent expression of AMPs (Levy *et al*, 2015). Thus, the gut microbiota acts both up‐ and downstream of the NLRP6 inflammasome, and the reciprocal regulation between the gut microbiota and the NLRP6 inflammasome plays a central role in the fine‐tuning of the gut homeostasis.

Of note, the impact of the NLRP6 inflammasome on the composition of the gut microbiota draws controversy. Two different groups have reported that NLRP6 deficiency does not influence the composition of the gut microbiota (Lemire *et al*, 2017; Mamantopoulos *et al*, 2017) (Fig 3). In these studies, the microbiota of *Nlrp6* ^−/−^ mice and littermate controls were compared, whereas earlier studies used non‐littermate controls (Elinav *et al*, 2011; Levy *et al*, 2015). Clearly, the impact of the NLRP6 inflammasome on the gut microbiota depends on the pre‐existing community structure in the respective vivarium.

## AIM2 inflammasome

AIM2 was first identified as a gene that was lacking in melanoma cell lines using subtractive cDNA hybridization (DeYoung *et al*, 1997). AIM2 protein is a member of the PYHIN protein family that is characterized by an N‐terminal PYD and one or two C‐terminal HIN (hematopoietic expression, IFN‐inducible nature, nuclear localization) domains (Unterholzner *et al*, 2010). AIM2 is a non‐NLR protein that is capable of forming an inflammasome complex. AIM2 recognizes and directly binds to cytosolic dsDNA (viral, bacterial, mammalian, and synthetic) via its HIN domain and recruits ASC to activate caspase‐1 (Burckstummer *et al*, 2009; Fernandes‐Alnemri *et al*, 2009; Hornung *et al*, 2009; Roberts *et al*, 2009). Although the lack of sequence specificity makes self‐DNA a potential ligand for AIM2, the AIM2 inflammasome is an essential sensor for microbial pathogens that invade the cytosol (Fernandes‐Alnemri *et al*, 2010; Rathinam *et al*, 2010; Sauer *et al*, 2010).

Like other inflammasome proteins, the AIM2 inflammasome has a physiological function to control the gut microbiota (Fig 3) (Hu *et al*, 2015; Ratsimandresy *et al*, 2017). Hu and colleagues reported that the AIM2 inflammasome senses microbial DNA derived from the commensal microbiota, leading to the secretion of IL‐18 and subsequent AMPs in the gut (Hu *et al*, 2015). The lack of AIM2 results in gut dysbiosis, thereby increasing the susceptibility to DSS‐induced colitis (Hu *et al*, 2015). The dysbiotic gut microbiota in mice deficient in AIM2 is accompanied by the expansion of colitogenic pathobionts, as the transmission of microbiota in *Aim2* ^−/−^ mice renders the co‐housed WT mice susceptible to colitis (Hu *et al*, 2015). In this context, the Hu and colleagues showed that an abundance of *Enterobacteriaceae*, including *E. coli*, was 1,000‐fold higher in feces collected from *Aim2*‐deficient mice compared to WT mice (Hu *et al*, 2015). Like NLRP6, the AIM2 inflammasome modulates the gut microbiota through the IL‐18–AMP axis (Levy *et al*, 2015). Consistent with this notion, *Aim2*‐deficient mice exhibit decreased levels of IL‐18 and AMPs, such as REG3γ and REG3β (Hu *et al*, 2015). Also, the administration of recombinant IL‐18 reduces the abundance of *E. coli* in *Aim2* ^−/−^ mice (Hu *et al*, 2015). Consistent with Hu laboratory study, Ratsimandresy and colleagues also proposed an alternative mechanism by which AIM2 controls the gut microbiota (Ratsimandresy *et al*, 2017). The Ratsimandresy laboratory demonstrated that increased populations of *Prevotella*, *Bacteroides,* and *mouse intestinal Bacteroides* (MIB) in *Aim2* ^−/−^ mice compared to WT mice, confers the susceptibility to colitis on the host. To determine a mechanism, they focused on the role of IL‐18 in the regulation of IL‐22 signaling. IL‐22 is known to regulate the expression of AMPs in intestinal epithelial cells, including REG3γ and REG3β (McDonald *et al*, 2006; Hill *et al*, 2013; Manta *et al*, 2013). IL‐18 inhibits IL‐22 binding protein (IL‐22BP), a soluble IL‐22 receptor protein that antagonizes the effect of IL‐22 signaling, and whereby AIM2‐mediated IL‐18 secretion reinforces IL‐22 signaling via IL‐22BP inhibition. Collectively, these observations indicate the AIM2 inflammasome has a function to maintain an intestinal microbial homeostasis through the production of IL‐18‐mediated AMPs in the gut. Note, however, the use of non‐littermate rather than littermate control mice in the studies conducted by Hu and Ratsimandresy and their colleagues (Hu *et al*, 2015; Ratsimandresy *et al*, 2017).

## Concluding remarks

The genetic mutations in inflammasomes are linked to autoinflammatory disorders. As inflammasomes are a key regulator underlying chronic diseases driven by inflammation that often arise in aging populations living a Western lifestyle, their physiological role has earned more attention among investigators. Although a primary function of inflammasomes is as an elaborate sensor, enabling a host to discriminate beneficial bacteria from malicious bacteria, inflammasomes are also the intermediaries in the cross talk between a host and its intestinal inhabitants. As discussed in this review, the environmental condition of the intestinal lumen is constantly translated into the host response to induce specific signals through IL‐1β or IL‐18 production, which results in a modulation of the intestinal microbiota by IL‐22 and AMPs. In turn, the modulated microbiota may enhance the host response through microbial metabolites, such as short‐chain fatty acids and bile acid derivatives. Thus, inflammasomes are indispensable in their ability to coordinate a precise reciprocal interaction in the body. On the other hand, the commensal microbiota should be regarded as one of the most important factors contributing to IBD pathogenesis. Our review of studies involving inflammasome mutant mice provided evidence that inflammasome activation through exposure to the normal commensal microbiota can exacerbate an intestinal inflammation if the intestinal epithelial integrity is impaired, or if the genetic background of the host contributes some influence. Considering that IBD develops when genetically susceptible individuals with impaired epithelial function are exposed to a trigger, such as the intestinal microbiota, the studies of inflammasome function suggest that inflammasomes recapitulate the actual clinical picture that occurs in IBD patients.

Data obtained from mouse models provide valuable insight, increasing the understanding of the association between the inflammasome, intestinal microbiota, and disease. In the field of gastroenterology, accumulated evidence using mouse models elucidates the potential pathogenic link between the inflammasome and IBD. Despite contradictory findings, it is likely that the aberrant activation of the inflammasome worsens IBD pathogenesis. Thus, inhibitors of the inflammasome and its downstream mediators, such as IL‐1β and IL‐18, may be optimal targets for the treatment of IBD. On the other hand, the inhibition of the inflammasome may have no effect or even a negative impact in individuals who lack increased expression of inflammasome‐related pathways. Given the ability of the inflammasome to prevent the expansion of pathobionts, inhibition of the inflammasome may exacerbate disease. Thus, in the clinical setting, the identification of patients who may respond to inflammasome‐targeted therapies may be critical.

**Pending issues**

- We do not have concrete evidence of NLRP1 inflammasome function to modulate the intestinal microbiota, nor do we know the clinical impact of the NLRP1 inflammasome on disease.
- Although a genetic study has identified a SNP associated with ulcerative colitis, it remains unclear what proportion of patients can be associated with the dysregulation of the NLRP3 inflammasome with regard to IBD pathogenesis.
- Although gain‐of‐function mutations of the NLRC4 gene cause autoinflammation with infantile enterocolitis, the role of the NLRC4 inflammasome in IBD remains uncertain.
- There is some evidence that refutes the impact of the NLRP6 inflammasome on the intestinal microbiota.
- Clinical relevance between the AIM2 inflammasome and IBD pathogenesis remains unclear.

## Author contributions

DW, YG, and NK wrote the manuscript.

## Conflict of interest

The authors declare that they have no conflict of interest.
